# Anatomic Criteria Determine Resectability in Locally Advanced Pancreatic Cancer

**DOI:** 10.1245/s10434-021-10663-1

**Published:** 2021-08-27

**Authors:** Georgios Gemenetzis, Alex B. Blair, Minako Nagai, Vincent P. Groot, Ding Ding, Ammar A. Javed, Richard A. Burkhart, Elliot K. Fishman, Ralph H. Hruban, Matthew J. Weiss, John L. Cameron, Amol Narang, Daniel Laheru, Kelly Lafaro, Joseph M. Herman, Lei Zheng, William R. Burns, Christopher L. Wolfgang, Jin He

**Affiliations:** 1. Department of Surgery, Johns Hopkins University School of Medicine, Baltimore MD, USA; 2. Department of Surgery, Queen Elizabeth University Hospital, Glasgow, Scotland, UK; 3. Department of Surgery, Nara Medical University, Nara, JP; 4. Department of Surgery, University Medical Center Utrecht, Utrecht, the Netherlands; 5. Department of Radiology, Johns Hopkins University School of Medicine, Baltimore MD, USA; 6. Department of Pathology, The Sol Goldman Pancreatic Cancer Research Center, Johns Hopkins University School of Medicine, Baltimore MD, USA; 7. Department of Surgery, Northwell Health, Manhasset NY, USA; 8. Department of Radiation Oncology, Johns Hopkins University School of Medicine, Baltimore MD, USA; 9. Department of Oncology, Johns Hopkins University School of Medicine, Baltimore MD, USA; 10. Department of Radiation Oncology, Northwell Health, Manhasset NY, USA; 11. Department of Surgery, New York University, New York, NY, USA

## Abstract

**Background:**

The introduction of multi-agent chemotherapy and radiation therapy has facilitated potential resection with curative intent in selected LAPC patients with excellent outcomes. Nevertheless, there remains a remarkable lack of consensus on the management of LAPC. We sought to describe the outcomes of patients with LAPC and objectively define the multidisciplinary selection process for operative exploration based on anatomical factors.

**Methods:**

Consecutive patients with LAPC were evaluated in the multidisciplinary clinic of a high-volume institution for pancreatic surgery between 2013 and 2018. Prospective stratification (LAPC-1, LAPC-2, and LAPC-3), based on the involvement of regional anatomical structures, was performed at the time of presentation prior to initiation of treatment. Resection rates and patient outcomes were evaluated and correlated with initial anatomic stratification system.

**Results:**

Overall, 415 patients with LAPC were included in the study, of whom 84 (20%) were successfully resected with a median overall survival of 35.3 months. The likelihood of operative exploration was associated with the pre-treatment anatomic LAPC score with a resection rate of 49% in patients classified as LAPC-1, 32% in LAPC-2, and 11% in LAPC-3 (*p*<0.001). Resected patients with improvement of the LAPC score at the time of exploration had significantly longer median overall survival as compared to those with no change or progression of LAPC score (60.7 vs 29.8 months, *p*=0.006).

**Conclusions:**

Selected patients with LAPC can undergo curative-intent surgery with excellent outcomes. The proposed Johns Hopkins anatomic LAPC score provides an objective system to anticipate the probability of eventual surgical resection after induction therapy.

## INTRODUCTION

Pancreatic ductal adenocarcinoma (PDAC) has low survival rates with early metastases^1,2^. In patients who present with localized disease, surgical resection of the primary tumor combined with multimodality therapy improves survival, particularly when negative margins are obtained^3^. Under this prism, the concept of borderline resectable (BRPC) disease was evolved for a subgroup of patients in whom the relationship of the tumor with major vascular structures makes a margin-negative resection with upfront surgery unlikely; these patients appear to benefit from neoadjuvant therapy. The heterogeneity in PDAC characterization as BRPC indicates that resectability is less defined by distinct categories and more accurately represented by an evolving conditional spectrum of surgical and biological variables^4,5^. This has practical implications since tumor misclassification may eliminate a subset of patients from consideration of a potentially curative operation.

For Stage III locally advanced pancreatic cancer (LAPC) current guidelines recommend a multidisciplinary approach, but ultimately declare non-operative systemic therapy based on patient performance status as definitive treatment^6^. The introduction of more aggressive multi-agent induction chemotherapy and radiotherapy has created a potential for curative resection in patients with LAPC, leading to improved survival^7–10^. Nevertheless, there is a distinct lack of an evidence-based consensus for the multidisciplinary management for LAPC patients resulting in a wide variation in treatment attitudes and preferences, including the propensity to consider exploration^11,12^.

An important driver of the variation in the management of LAPC lies partially in the lack of a widely used paradigm to identify patients with LAPC who may be eligible for surgical exploration and resection after induction therapy. As an initial step in developing a consensus, the goal of this study was to report the use of the Johns Hopkins LAPC score based off of objective anatomic criteria and perceived likelihood for surgical resection in patients with LAPC. Second, we support the validity of these selection criteria based on perioperative outcomes and disease specific survival. To accomplish this, all patients with LAPC who were reviewed in the Johns Hopkins Pancreatic Cancer Multidisciplinary Clinic (PMDC) were analyzed.

## METHODS

### Patient cohort

All patients who presented with a new diagnosis of PDAC at the Johns Hopkins PMDC from January 2013 to March 2018 were eligible for this study (n=823). The National Comprehensive Cancer Network (NCCN) guidelines were utilized for initial LAPC definition^13^. PMDC patients with initial classification of resectable, BRPC, or metastatic PDAC were excluded from the study. Resection eligibility was assessed based on standardized pancreatic protocol CT with 3-dimensional angiogram; patients evaluated with out-of-institution imaging only were excluded for cohort homogeneity. At the time of first evaluation, patients with LAPC were prospectively assigned into three tiers based on imaging findings and the perceived likelihood to undergo surgical exploration and resection after completion of induction therapy: LAPC-1 (likely), LAPC-2 (unlikely), and LAPC-3 (highly unlikely). The stratification was performed at the time of initial presentation by the consensus of a multidisciplinary panel led by experienced pancreatic surgeons. Vessel abutment was defined as contact ≤180°, and encasement as >180° contact on preoperative imaging.

Patients were routinely reviewed during induction therapy at PMDC in 2-month intervals for treatment response assessment and reconsideration of tumor resectability. Those who developed distant metastasis or suffered deteriorating performance status were considered ineligible for surgical exploration. Local tumor progression, response to induction chemotherapy based on RECIST (Response Evaluation Criteria in Solid Tumors^14^) criteria, and serum CA19–9 were important factors guiding multidisciplinary treatment decisions (i.e. surgical exploration versus additional induction therapy. However, a subset of patients without a radiographic response or even local tumor during induction therapy were considered for surgical exploration due to previously established limitations of defining fibrosis versus tumor in post-treatment imaging^15^. Diagnostic laparoscopy was routinely performed on the day of planned resection with any suspicious lesions biopsied and sent for frozen section prior to open exploration. Peritoneal washings were not performed. Resection rates for each tier were identified and patient outcomes subsequently assessed. This study was approved by the institutional review board. For the purpose of this study, all patients who underwent surgical exploration had their LAPC score retrospectively reassessed as determined by preoperative imaging performed at the completion of neoadjuvant therapy. Three classifications were defined: progression, no change or improvement of initial LAPC score with the additional inclusion of resectable or borderline disease defined by NCCN guidelines (Supplemental Figure 1).

### Data collection

PMDC disposition notes and the prospectively maintained institutional pancreatectomy database were utilized for collection of patient demographic data. Imaging data focused on tumor size and site, tumor involvement of the gastrointestinal tract and/or major vessels. Systemic induction therapy was documented, including type and duration of chemotherapy regimens, radiotherapy modalities, and treatment changes or complications, where applicable. In patients with LAPC who underwent surgical resection, we further assessed perioperative data and postoperative outcomes. Pathology features, including resection margin status, were extracted from final pathology reports. Identification of invasive carcinoma within 1mm (≤1mm) of the surgical margin was characterized as R1. All follow-up data were documented until patient death, or date of last follow-up.

### Definitions, statistical analysis, and algorithm development

The Mann–Whitney *U* test and *χ*^*2*^ test were utilized for the analysis of continuous and categorical variables, respectively. Overall survival (OS) was defined as the time between date of diagnosis and either death or last follow-up. Recurrence-free survival (RFS) was defined as the time between date of surgery and either date of recurrence or last follow-up if recurrence was not observed. Median patient survival was estimated using Kaplan–Meier curves with corresponding 95% confidence intervals (CIs). Log-rank tests were utilized for survival comparisons. Statistical significance was defined by a 2-sided *p* value of <0.05. Statistical analysis was performed with the SPSS statistical software version 25.0 (IBM Corporation, Armonk, NY, USA).

Initial LAPC score was assigned prospectively based on the routine approach of patient imaging at PMDC: identification of 1. aberrant anatomy, 2. arterial involvement, 3. venous involvement, and 4. presence of collateral veins within the potential field of resection. Patients of different vessel involvement combinations were categorized between the three tiers. All imaging scans were reevaluated retrospectively to confirm the anatomic criteria inherent to the algorithm.

## RESULTS

### Patient cohort and characteristics

**Figure 1:**
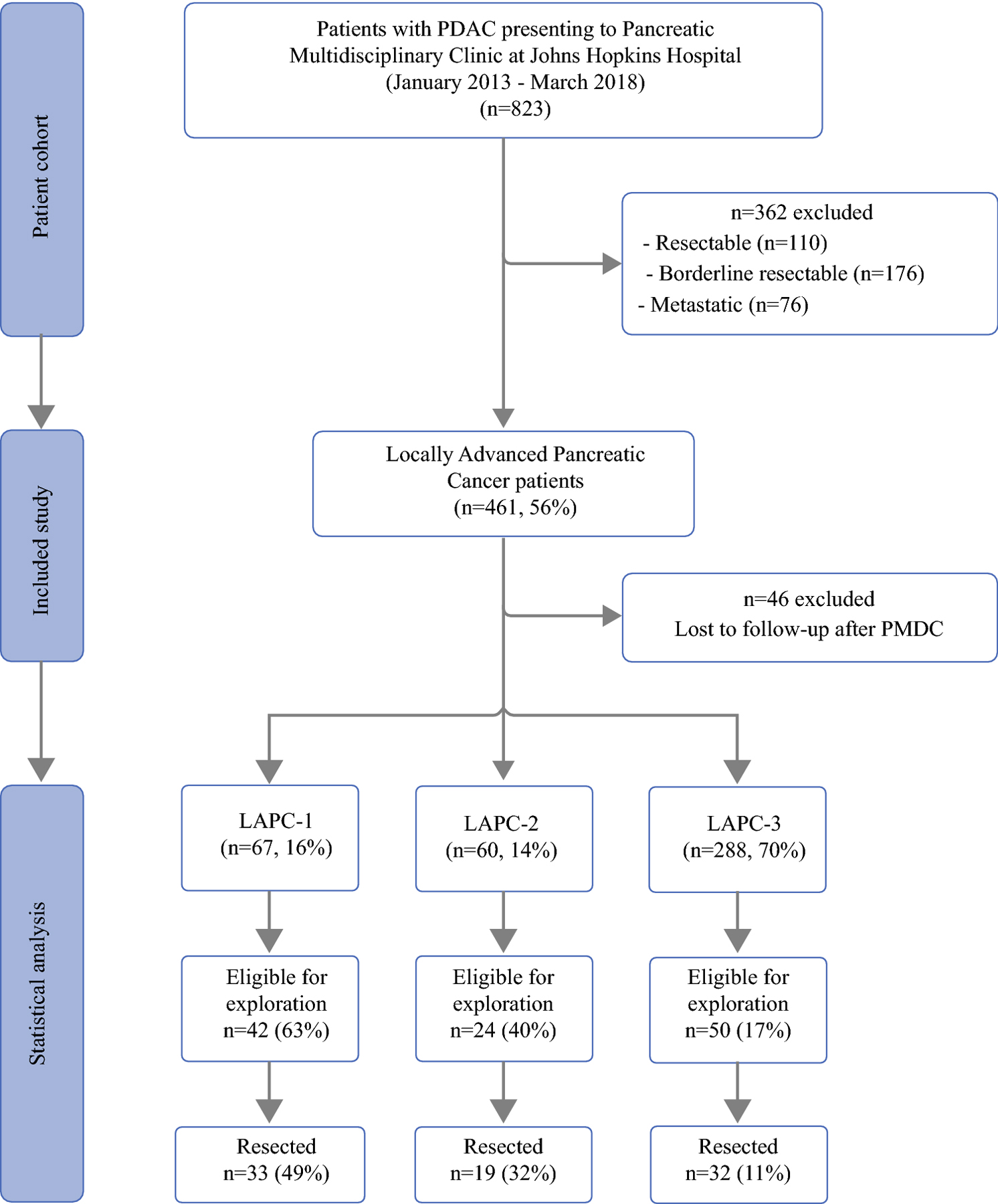
Patient study selection flowchart

Overall, 823 patients with PDAC were reviewed at the institutional PMDC within the studied period. 461 patients with LAPC were identified (56%); they were all recommended to have induction chemotherapy and/or chemoradiation and were re-evaluated routinely for assessment of treatment response. Forty-six patients (10%) were lost to follow-up and excluded from the final analysis (Figure 1). The remaining 415 patients with LAPC were stratified into one of three scores at the time of initial presentation by the consensus of a multidisciplinary panel led by experienced pancreatic surgeons. This included 67 (16%) classified as “likely” to undergo resection or LAPC-1, 60 (14%) as “unlikely” or LAPC-2, and 288 (70%) as “highly unlikely” or LAPC-3. Demographic characteristics were comparable between groups (Table 1).

Most LAPC patients received induction chemotherapy (LAPC-1 97%, LAPC-2 87%, LAPC-3 91%, *p*=0.11) with FOLFIRINOX-based regimens the most frequently utilized (51% of LAPC-1, 42% of LAPC-2, and 45% of LAPC-3, *p*=0.30). The median duration of induction chemotherapy was between 4 to 5 months for all three classification groups (*p*=0.69). Stereotactic body radiation therapy (SBRT) was the modality of choice for induction radiation treatment (60% in LAPC-1, 57% of LAPC-2, and 30% in LAPC-3, *p*=0.03).

When comparing between stratification groups, patients with LAPC-3 had larger tumors (p=0.004) and were more likely to involve the gastrointestinal tract (p=0.03) than LAPC-1 and LAPC-2 scores. Baseline CA19–9 levels were not significantly different among the three tiers (*p*=0.43). Multiple combinations of vessel abutment and/or encasement were identified and described with higher rates of vessel encasement in patients with LAPC-3 compared to LAPC-1 and LAPC-2 scores (Table 1).

### Resection rates and perioperative outcomes

The decision for surgical exploration was based primarily on performance status and lack of distant disease progression while also considering tumor response to chemotherapy. After ongoing evaluation, 116 patients with LAPC were deemed eligible for surgical exploration (28%). Notably, exploration rates were significantly different between the prospectively defined LAPC tiers: 63% in LAPC-1, 40% in LAPC-2, and 17% in LAPC-3 (Figure 1). Consequently, successful resection of the primary tumor was achieved in 33/67 LAPC-1 (49%), 19/60 LAPC-2 (32%), and 32/288 LAPC-3 patients (11%, *p*<0.001). Exploration without successful resection occurred in 32 patients with occult metastatic disease identified in 18 patients (56%) and incomplete or aborted resection in the remaining 14 patients (44%); extensive arterial invasion was the most common reason for failed resection and eight patients underwent irreversible electroporation. When comparing initial LAPC scores to a re-classification score based on preoperative imaging in patients that underwent exploration, 37% had improvement, 6% had progression and 57% had no change of LAPC score.

No differences in intraoperative characteristics and perioperative outcomes were associated with LAPC score (Table 2). Patients across all groups most often underwent a pancreaticoduodenectomy (52%) or a distal pancreatectomy with celiac artery resection (DP-CAR, 27%, *p*=0.18). Vein resection rates were comparable (*p*=0.71), whereas no SMA resections were performed. The occurrence of any postoperative complication was seen in 54% of all patients, however a Clavien-Dindo grade ≥ IIIb was only observed in 5% of patients. No differences were identified in estimated blood loss (*p*=0.16), length of stay (*p*=0.43), postoperative 30-day morbidity (*p*=0.75), and 90-day mortality (*p*=0.15).

A complete negative-margin resection was obtained in 88% of patients. R0 resection rates were identical among the three LAPC scores (88–89%, *p*=0.76). Additionally, most patients among the three scores had extensive (grade 1) or moderate (grade 2) response to induction chemotherapy (62–81%) based on CAP score^16^.

### LAPC classification algorithm

**Figure 2:**
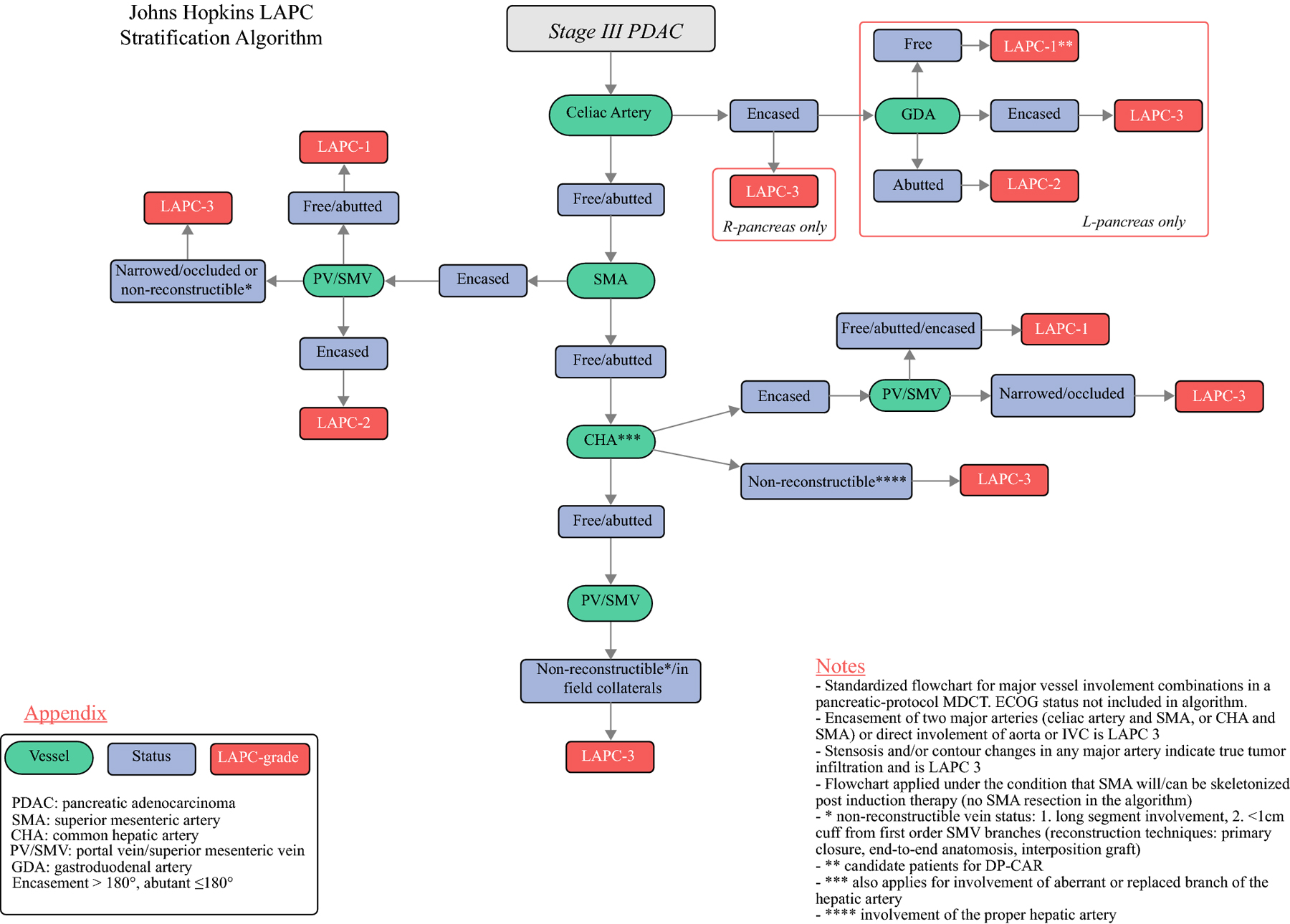
The Johns Hopkins LAPC score algorithm

The algorithm for the categorization of patients with LAPC in three score tiers is shown in Figure 2 and Table 3. Like the established BRPC classification, vascular involvement is assessed in a stepwise fashion when viewing preoperative pancreatic protocol CT starting with the three major arterial structures: celiac artery (CA), superior mesenteric artery (SMA), and common hepatic artery (CHA). This initial assessment of arterial involvement forms the “trunk” of the flow chart with “branch” clusters determining the nuanced anatomic criteria in each LAPC patient as directed by the combination of gastroduodenal artery (GDA) and portal vein (PV)/superior mesenteric vein (SMV) involvement.

**Figure 3:**
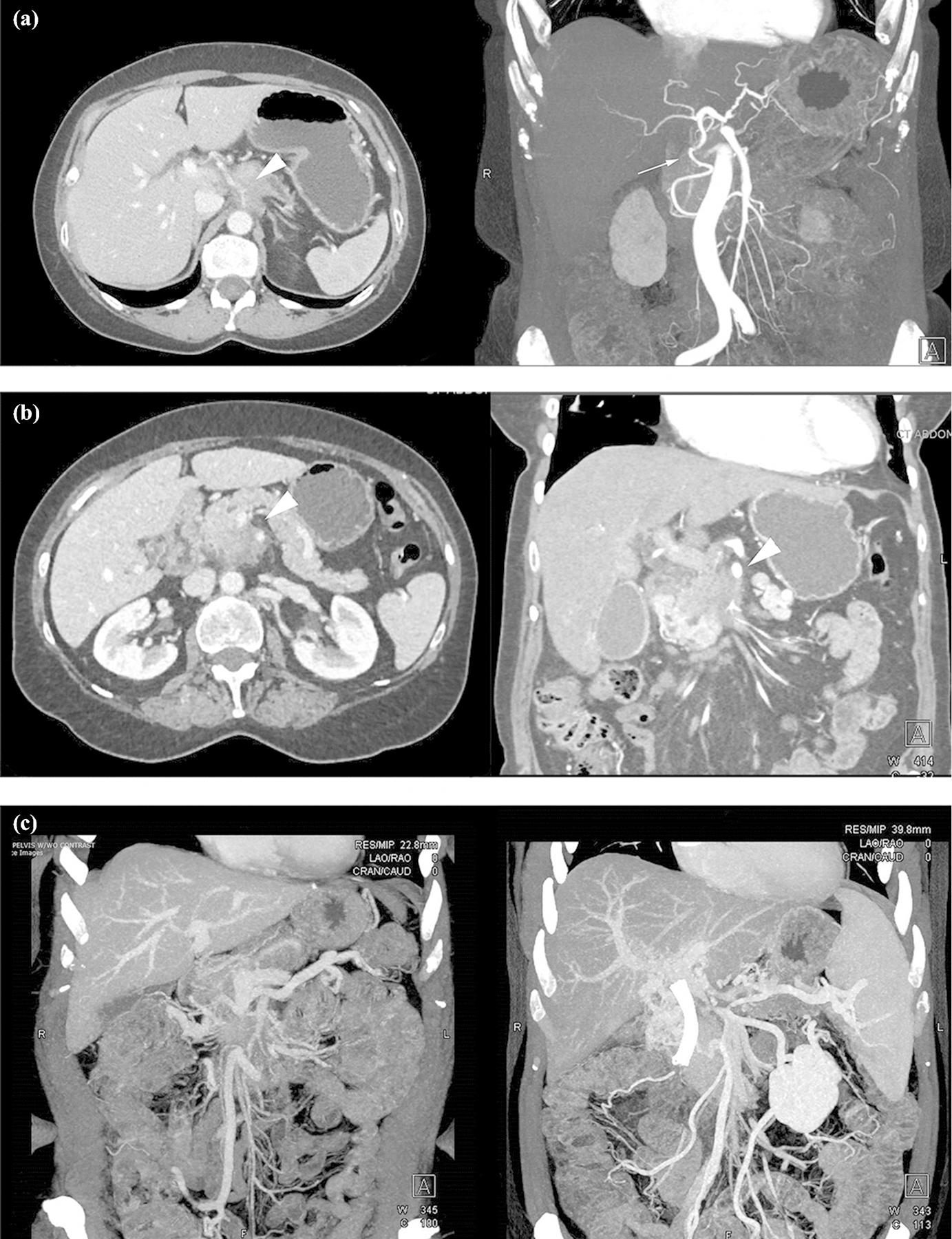
(a) CT imaging of a patient with LAPC-1: encasement of the celiac artery (arrowhead) and no involvement of the gastroduodenal artery (arrow), (b) CT imaging of a patient with LAPC-2: encasement of the SMA and SMV (arrowheads), (c) CT imaging of a patient with PDAC and non-reconstructible SMV, LAPC-3.

Evaluation of involvement of the CA by the tumor was the first step in determining LAPC score. In right-sided pancreatic lesions, encasement of the CA was scored as LAPC-3 and “highly unlikely” to undergo exploration after induction therapy, since surgical options would require resection of both CA and the GDA, precluding a DP-CAR. In left-sided tumors, classification was based on the status of the GDA and the probability of performing a DP-CAR (Figure 3a). The second step was to evaluate SMA and CHA tumor involvement. In patients with SMA encasement, the LAPC score was determined by the concomitant PV/SMV involvement (Figure 3b). In patients with CHA involvement, any concomitant changes in PV/SMV contour (narrowing or occlusion) were stratified as LAPC-3; additionally, isolated long CHA segment involvement that necessitated resection and anastomosis at the level of the proper hepatic artery (PHA) was deemed LAPC-3. This stratification also applied in patients with encasement of an aberrant or replaced hepatic artery.

If all major arteries were free or abutted by the lesion, LAPC score was based on the status of the PV/SMV. In cases with (1) encasement of a long vein segment, (2) involvement of SMV within less than 10mm from the first-order tributaries, or (3) cavernous transformation in proximity to the tumor the vein was deemed non-reconstructible and deemed LAPC-3 (Figure 3c). Additional LAPC-3 score representations included patients with direct involvement of the aorta or the inferior vena cava, encasement of two major arteries (SMA and CA or SMA and CHA), and artery contour changes that indicated tumor infiltration. The total distribution of determining anatomic criteria for every case of LAPC classification can be found in Table 3.

### Disease recurrence and survival

**Figure 4:**
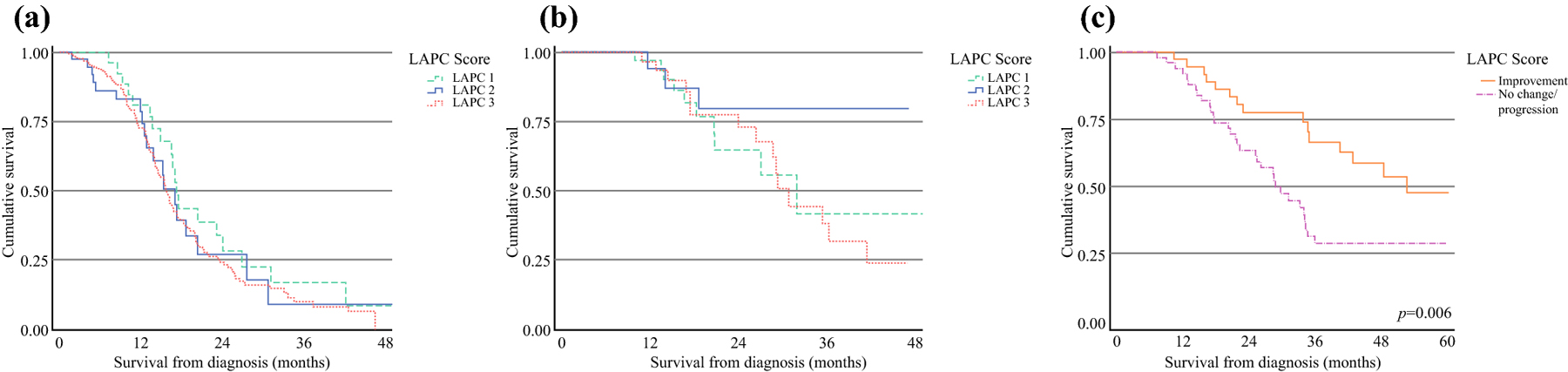
Overall survival in patients with pancreatic cancer stratified by preoperative LAPC-score for (a) unresected and (b) resected patients, p>0.05. (c) Overall survival of resected patients with LAPC comparing those with improvement of their initial LAPC score after neoadjuvant to those with no change or progression; p=0.006.

At last follow-up, 48% of the resected patients had presented with disease recurrence of similar distribution among the tiers (*p*=0.11). Patients with LAPC who underwent surgical resection had significantly longer median overall survival compared to patients who received systemic therapy alone (35.3 vs. 16.2 months, *p*<0.001). When comparing the three LAPC score groups, patients who were not resected due to disease progression or poor performance status all had comparable median overall survival: 17.5 months for LAPC-1, 17 months for LAPC-2, and 15.8 months for LAPC-3 (*p*=0.14). In the resected cohort, median overall survival was not reached for LAPC-1 and LAPC-2, whereas patients with LAPC-3 had a median survival of 30.7 months (Figure 4a, b). Notably within this resected cohort, patients with improvement of LAPC score had significantly longer median overall survival compared to patients who had progression or no change of score from the time of diagnosis to preoperative imaging (60.7 vs 29.8 months, *p*=0.006) (Figure 4c).

## DISCUSSION

**Figure 5:**
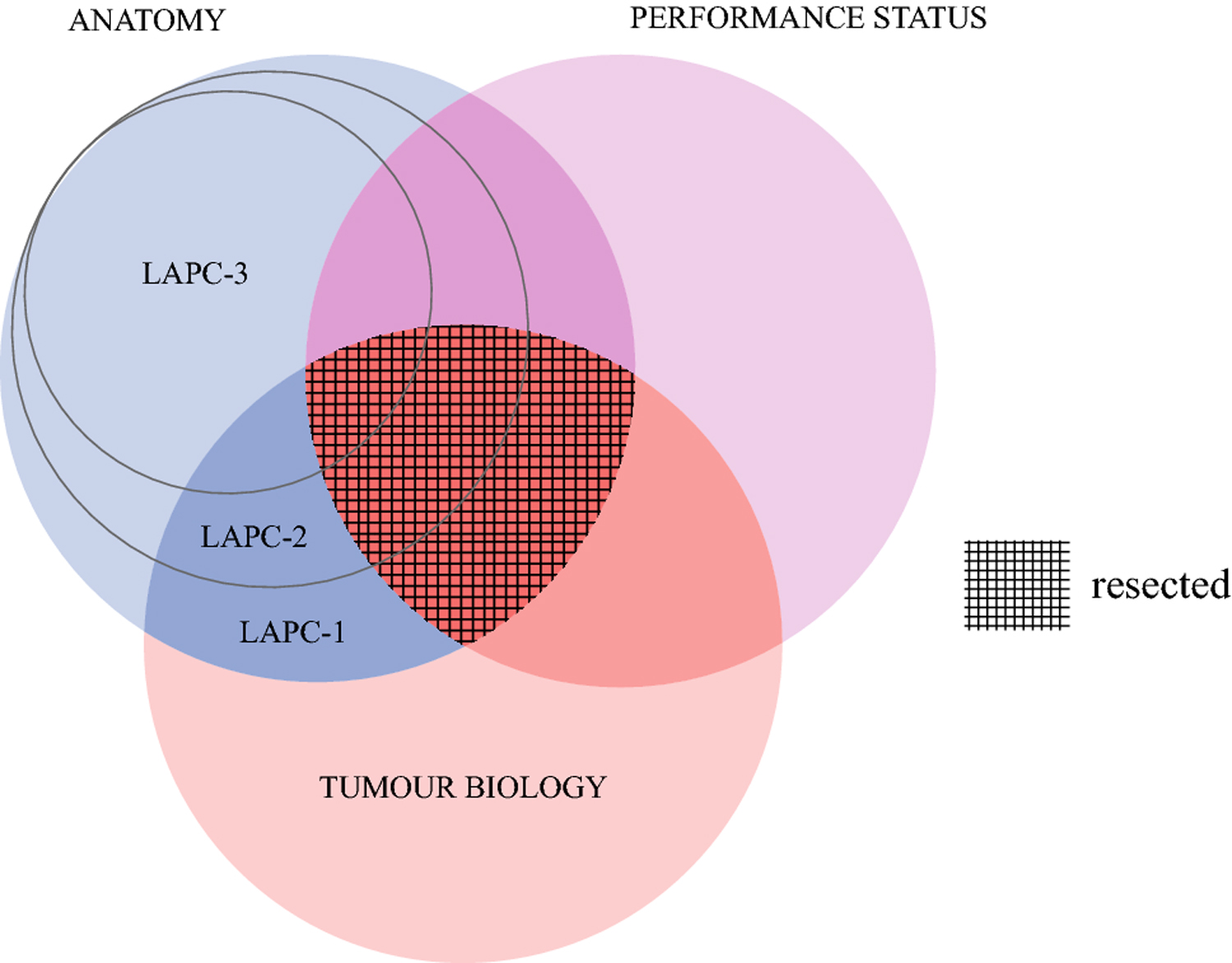
Venn diagram of resectability in locally advanced pancreatic cancer as a combination of anatomy, patient performance status and tumor biology. Different LAPC tiers correspond to different portions of potentially resectable patients.

Improved survival of patients with localized PDAC is currently best achieved through resection of the tumor with grossly negative margins^17^. In LAPC, this translates into a risk/benefit balance between achieving a safe resection in the context of preservation or successful reconstruction of major vasculature and performing a non-therapeutic laparotomy with inherent risks. This was the drive behind the development of the concept of BRPC and was brought to the forefront with the introduction of neoadjuvant therapy^18,19^. Different groups have proposed variable definitions^20^ using anatomical, biological, and conditional parameters for BRPC^4^. This study shows that patients with tumors defined as LAPC represent a broad group with significant variance in anatomical involvement. Careful patient selection and successful tumor extirpation within this broad subset results in excellent long-term survival outcomes compared to systemic therapy alone. The novel prospective LAPC classification system proposed in this manuscript is effective in helping to identify the patients who are more likely to proceed with successful surgical intervention within this cohort classically deemed “unresectable”. Thus, like BRPC the resectability in patients with LAPC is also defined by the same criteria: tumor anatomical extent, biological behavior, and patient performance status (Figure 5).

Previous groups have also identified the lack of differentiation within the large LAPC cohort and have similarly attempted to develop an additional scoring system. Fromer et al. performed a systematic review of patients with LAPC disease and underwent pancreatectomy with different associated vascular resections. Additional staging was recommended according to arterial involvement: Stage IIIa as celiac or common hepatic only arterial involvement, IIIb1 as SMA only involvement, Stage IIIb2 as celiac and SMA involvement and Stage IIIc as non-reconstructible venous involvement^21^. Of note, the analysis by Fromer et al. is based on review of patients undergoing pancreatectomy with vascular resection. Therefore, the study does not capture the total “n” of LAPC patients and likely misses a cohort of LAPC patients undergoing surgical resection without arterial resection (i.e. those who underwent SMA divestment and did not require arterial reconstruction). In contrast, the anatomic LAPC staging proposed by our manuscript represents a score based on pretreatment imaging. By including patients that fail to undergo exploration, there is a more comprehensive estimate of the likelihood for surgical resection. Furthermore, the prospective capture of LAPC patients within a single institution with the same set of high-volume pancreatic cancer surgeons involved in both the preoperative evaluations and surgical resections limits some biases inherent in literature review pooled across multiple institutions and 20 years of practice. Chatzizacharias et al. similarly proposed an additional LAPC staging system based on vascular involvement^22^. This is an important contribution from an experienced group of pancreatic surgeons and highlights the value of subclassifying LAPC based on the likelihood of surgical resection. Our manuscript offers a larger cohort of patients (415 vs 96) while also uniquely noting the importance of combined vascular and venous involvement. While reconstructible venous involvement alone is considered borderline resectable disease, its combination with arterial involvement can complicate resection and may reflect a more invasive biology. Based on our staging system, SMA encasement alone without venous involvement represented 55% of our LAPC 1 patients, while SMA encasement with concomitant reconstructible SMV/PV involvement represent 73% of LAPC 2 in this study and finally SMA encasement with non reconstructible SMV/PV is classified as LAPC 3. In the present proposed scale, involvement of CHA is similar: CHA encasement with concomitant reconstructible venous disease is scored as LAPC 1; CHA encasement with non reconstructible venous disease is scored as LAPC 3. The flow chart based staging system approach considering the compounding effects of all vascular involvement offers additional granularity and an important distinction when elucidating the preoperative probabilities of a patient completing therapy and undergoing successful tumor extirpation. Furthermore, our study identified an association between improvement in LAPC substage during induction therapy and overall survival, highlighting its potential for both predictive and prognostic value.

The proposed algorithm demonstrates a standardized surgical decision flowchart for assessment of potential future LAPC resectability. We observed concordance with surgical exploration rates and prospective LAPC tier classification. Most patients were classified with a LAPC-3 score (70%), indicating that regardless of a favorable response to induction therapy, there were limited surgical options for successful resection using current widely accepted techniques. These patients had significantly larger tumors at diagnosis and were more likely to present with encasement of major arteries. The disparities in anatomical criteria were reflected in exploration and resection rates as almost half of the patients with LAPC-1 were eventually resected, compared to one third of LAPC-2 and 10% of LAPC-3.

In LAPC-1 patients, surgical exploration and resection rates are 63% and 49%, respectively. A recent report from our institution demonstrates a 64% resection rate in BRPC after neoadjuvant treatment^23^. Further comparison of overall survival also shows similar patient outcomes (>29 months for LAPC-1 vs. 28.8 months in BRPC). These data indicate that tumor resectability is represented more accurately by a continuum spectrum, a “grey-zone” that shifts towards more complex LAPC with the development of effective chemotherapy regimens^6,24^. This is highlighted by the debate regarding eligible patients for DP-CAR, who are evenly considered as BRPC and LAPC. The evolution of multi-modality treatments^25^ and the increasing complexity of pancreatic surgery in high-volume centers demonstrate the necessity for objective identification of patients with LAPC who are eligible for resection based on anatomic criteria, and continuous reassessment of BRPC and LAPC terminology. However, the definitive aspect of tumor resectability in LAPC is tumor biology. There is an *a priori* selection bias, due to the inability to predict the biological behavior of the tumor. Currently we offer universal systemic treatment to all LAPC patients, without knowing if and how they will respond, rendering potential future resection primarily a matter of anatomic fortuity.

A variety of surgical approaches was utilized for patients with LAPC who were explored. While pancreaticoduodenectomy was the most frequently employed, distal pancreatectomy with celiac axis resection (DP-CAR) was the second most common operation (27%). The surgical rationale in DP-CAR is that liver arterial supply is preserved via retrograde flow and therefore lack of GDA involvement by the tumor is mandatory for this operation^26^. Compromise of the GDA significantly decreases resection potential (LAPC-2 or −3) and it is our opinion that GDA status must be specifically addressed on imaging reports to determine resectability potential. Involvement of the CHA was also common in the studied cohort. For most patients, isolation of the CHA proximal to GDA was possible, since true invasion by the tumor is uncommon and a dissection plane can be identified^27–29^.

In presence of SMA encasement, patients were explored with an “artery-first” approach to identify the extension of arterial involvement^30–33^. SMA divestment was accomplished with a perivascular tissue clearance in the plane between the adventitia and the perineural sheath^34–36^. There are emerging retrospective reports regarding favorable outcomes in patients who undergo SMA resection^37–39^, however, this is not a technique performed in our practice with arterial divestment instead universally pursued. More data are needed to justify SMA reconstruction, that often entails high perioperative morbidity and mortality^40,41^. The status of the PV/SMV was the final major regulator. Vein resection was performed in approximately 50% of patients. Reconstruction techniques included primary vein anastomosis, vein patch, or interposition graft placement after segmental resection^42,43^. Prolonged occlusion of the PV/SMV by the tumor often resulted to extensive venous collateralization. Preservation of left-sided collaterals occasionally allowed surgical resection without a need for venous reconstruction^44^.

All patients in this study were followed with clinical assessments, standardized interval CT imaging and follow-up of CA19–9; these were factored into clinical decisions. Yet, it remains to be determined what constitutes the optimal modality and the most credible biomarker for assessment of treatment response. Imaging is the basis for anatomical evaluation of the tumor, but further studies are needed to identify if it is a reliable predictor of resectability after preoperative therapy^45^. Furthermore, CA19–9 is characterized by mediocre sensitivity and specificity but remains a cost-effective biomarker for patient surveillance^46,47^. Because of this, the practice of many high-volume institutions is to explore all LAPC patients with appropriate performance status after induction therapy if there is no sign of distant metastatic disease. The proposed novel LAPC classification, while defined solely by anatomic criteria, meaningfully reflects a component of tumor biology when viewed over time, as resected patients with improvement of their LAPC score at the time of operative exploration had significantly longer overall survival as compared to those with no change or progression of their anatomic LAPC score. With additional prospective study, the LAPC score and its change in response to induction therapy may prove to be a useful biomarker to better refine patient selection from the “explore all” paradigm. Initial induction treatment across this study was largely decided upon by the treating medical oncologist. Nevertheless, this stratification scale assisted in guiding multidisciplinary therapy. Patients classified as LAPC-1 based were more likely to undergo radiation therapy than the LAPC-3 cohort (76% vs 45%). Due to risks of gastrointestinal perforation, our group is more apt to utilize SBRT in patients more likely to undergo exploration, thus mitigating the perforation risk by resecting the duodenum as part of the specimen. Thus, this staging algorithm subdividing the broad LAPC group helps to guide the patient selection process and their treatments.

As multimodal therapy continues to improve and limits are pushed with advancing surgical technique, the concept of pancreatic cancer resectability is currently undergoing an ongoing evolution. With this, there are multiple reports of exceptional median overall survival in well-selected LAPC patients receiving multimodal systemic therapy and margin-negative resection ranging from 35 to >60 months^7,10,48^. Nevertheless, a lack of consensus in the management of LAPC persists underlying the importance of further subclassifying this broad cohort to objectively identify individuals more likely to undergo surgical exploration^11,12^. In this study we see that in selected patients margin negative resection rates approached 90% with a median overall survival of 35.3 months. This survival is enhanced even further to a remarkable 61 months in the cohort of resected patients with improvement from their prospective LAPC score compared to the LAPC score determined at preoperative imaging after neoadjuvant therapy.

This study has several limitations. First, albeit a prospective assignment of LAPC score, the retrospective investigation of correlative outcomes allows significant selection bias on different levels. Next, previous data have suggested that CT findings do not always correlate with “true” vascular involvement^15^. However, this is an inherent limitation consistent across all preoperative stratifications based on anatomic criteria. With this in consideration, our groups’ growing mentality is erring on the side of offering exploration in patients with biologically favorable disease, thus why many tumors classified as LAPC-2 or LAPC-3 with extensive vascular involvement still achieved R0 resections. This classification system is not to serve as a means of definitively excluding patients from exploration, moreover, it stratifies the antiquated term of “locally-advanced” disease to those that are most likely to achieve complete tumor extirpation. Additional limitations include the use of expert consensus as the foundation for the choice of induction therapy and the option for surgical exploration. Furthermore, while the proposed classification allows for a clinically accurate identification of patients with LAPC who may be eligible for resection, the operations are complex mandating experienced surgeons and substantial institutional resources and support during post-operative recovery to truly optimize patient outcomes.

## CONCLUSIONS

In this single-institution cohort study, carefully selected patients with LAPC underwent successful resection and achieved exceptional long-term survival. Prospective stratification of patients with LAPC at diagnosis in three different tiers based on anatomic criteria provides a valid basis for prediction of which patients are more likely to undergo future surgical exploration and potential resection of the primary tumor post induction therapy. The LAPC score is a valuable tool to expand the broad, evolving LAPC nomenclature and may provide a meaningful step towards consensus management. Even though this algorithm provides an objective assessment of resectability, patients with LAPC should be monitored closely and evaluated repeatedly during chemotherapy and explored in high-volume centers, since the surgical complexity of these cases has increased. Further prospective validation of this algorithm will provide additional information regarding its clinical utility.

## Supplementary Material

1741840_Sup_fig **Supplementary Figure 1:** Flow chart defining radiographic change in LAPC score after neoadjuvant therapy.

## Figures and Tables

**Table 1:** Demographics and clinical data of LAPC patients.

*Variable*	*LAPC patients (n=415)*	*LAPC-1 (n=67)*	*LAPC-2 (n=60)*	*LAPC-3 (n=288)*	*p*-value
Male, *n* (%)	195 (47)	33 (49)	26 (43)	135 (47)	0.623

Race, *n* (%)					0.367
Caucasian	348 (84)	62 (93)	52 (87)	236 (82)	

Age (years)					0.511
Mean (SD)	65.2 (10.1)	63.2 (9.7)	65.6 (9.9)	65.4 (10.7)	

ECOG performance status, *n* (%)					0.152
ECOG 0	131 (32)	26 (39)	23 (38)	82 (29)	
ECOG 1	260 (63)	39 (58)	36 (60)	185 (64)	
ECOG 2	21 (5)	1 (1)	1 (2)	19 (7)	
ECOG 3	3 (1)	1 (1)	0 (0)	2 (0)	

CT-scan characteristics, *n* (%)					
Tumour size (mm), mean (SD)	38 (14)	34 (13)	37 (12)	40 (15)	**0.004**
Regional lymphadenopathy, *n* (%)	111 (27)	14 (21)	13 (21)	84 (29)	0.177
GI tract involvement, *n* (%)	91 (22)	8 (12)	14 (23)	69 (24)	**0.039**
Duodenum	68 (75)	3 (4)	9 (15)	56 (19)	
Stomach	22 (24)	4 (6)	5 (8)	13 (5)	
Small bowel	1 (1)	1 (1)	0 (0)	0 (0)	
Tumour site, *n* (%)					0.288
Head	174 (42)	26 (39)	27 (45)	121 (42)	
Uncinate	62 (15)	10 (15)	6 (10)	46 (16)	
Neck	51 (12)	5 (8)	12 (20)	34 (12)	
Body	115 (28)	22 (33)	15 (25)	78 (27)	
Tail	13 (3)	3 (4)	1 (2)	9 (3)	
Atrophy of the pancreas, present, *n* (%)	258 (62)	36 (54)	36 (60)	186 (65)	0.090

Involvement of major vessels, *n* (%)					
Abutment (≤180°)					**0.008**
PV/SMV	70 (17)	19 (28)	18 (30)	33 (11)	
Celiac artery	35 (8)	9 (13)	12 (20)	14 (5)	
Superior mesenteric artery	68 (16)	17 (25)	19 (32)	32 (11)	
Common hepatic artery	21 (5)	5 (7)	5 (8)	11 (4)	
Encasement (>180°)					**<0.001**
PV/SMV	282 (68)	26 (39)	54 (90)	202 (70)	
Celiac artery	164 (40)	11 (16)	31 (52)	122 (42)	
Superior mesenteric artery	196 (47)	3 (4)	15 (25)	178 (62)	
Common hepatic artery	213 (51)	20 (30)	39 (65)	154 (53)	

CA19–9 at diagnosis ^a^					0.430
Median (IQR), U/ml	314 (66–1196)	195 (47–530)	325 (71–1220)	462 (102–1455)	

Induction chemotherapy, *n* (%)					0.304
FFX-based	189 (50)	34 (51)	25 (42)	130 (45)	
FFX-Gem combination	72 (19)	11 (16)	15 (25)	46 (16)	
Gem-based	117 (31)	20 (30)	12 (20)	85 (30)	
Time administered, months					0.694
Median (IQR)	4 (3–6)	5 (4–6)	5 (4–6)	4 (3–6)	

Induction radiotherapy, *n* (%)	222 (54)	51 (76)	41 (68)	130 (45)	**0.001**
Modality, *n* (%)					**0.036**
SBRT	160 (72)	40 (60)	34 (57)	86 (30)	
IMRT/standard RT	62 (28)	11 (16)	7 (12)	44 (15)	

Time from NAT to OR, weeks					0.745
Median (IQR)	6 (5–8)	6 (5–7)	6 (5–8)	6 (5–8)	

Surgical exploration, *n* (%)					**<0.0001**
Yes	116 (28)	42 (63)	24 (40)	50 (17)	
No	299 (72)	25 (37)	36 (60)	238 (83)	

OR outcome, *n* (%)					**<0.0001**
Resected	84 (20)	33 (49)	19 (32)	32 (11)	
Not-resected	331 (80)	34 (51)	41 (68)	256 (89)	

LAPC: locally advanced pancreatic cancer; SD: standard deviation; ECOG: Eastern Cooperative Oncology Group; CT: computed tomography; GI: gastrointestinal; PV: portal vein; SMV: superior mesenteric vein; IQR: interquartile range; CA19-9: Carbohydrate antigen 19-9; SBRT: stereotactic body radiation therapy; IMRT: Intensity-modulated radiation therapy; RT: radiotherapy; NAT: neoadjuvant therapy; OR: operating room; a: 67 missing values at the time of baseline MDT. Values in bold are statistically significant.

**Table 2:** Perioperative clinical and pathological data of resected LAPC patients.

*Variable*	*Resected LAPC patients (n=84)*	*LAPC-1 (n=33)*	*LAPC-2 (n=19)*	*LAPC-3 (n=32)*	*p*-value
Type of operation, *n* (%)					0.187
Whipple	44 (52)	16 (48)	12 (63)	16 (50)	
DP-CAR	23 (27)	13 (39)	3 (16)	7 (22)	
Distal pancreatectomy	16 (19)	4 (12)	3 (16)	9 (28)	
Total pancreatectomy	1 (1)	0 (0)	1 (5)	0 (0)	

Vein resection, *n* (%)	41 (49)	16 (48)	8 (42)	17 (53)	0.713

Diagnostic laparoscopy, *n* (%)	34 (41)	9 (27)	7 (37)	18 (56)	**0.017**

Estimated blood loss, ml					0.160
Median (IQR)	400 (250–1000)	400 (225–700)	350 (175–1100)	400 (250–1200)	
Length of hospital stay, days					0.437
Median (IQR)	8 (6–11)	7 (6–11)	8 (7–11)	8 (6–10)	

Morbidity, *n* (%)					0.755
≤ Clavien-Dindo grade IIIa	45 (54)	16 (48)	8 (42)	21 (66)	
≥ Clavien-Dindo grade IIIb	4 (5)	1 (3)	1 (5)	2 (6)	

Thirty-day readmission, *n* (%)	24 (29)	12 (36)	4 (21)	8 (25)	0.263
Ninety-day mortality, *n* (%)	3 (4)	0 (0)	1 (5)	2 (6)	0.158

Resection margin, *n* (%)^b^					0.768
R0	74 (88)	29 (88)	17 (89)	28 (88)	
R1	9 (11)	4 (12)	2 (11)	3 (9)	

Tumor differentiation, *n* (%)					0.499
Well	2 (2)	0 (0)	1 (5)	1 (3)	
Moderate	45 (54)	21 (64)	8 (42)	16 (50)	
Poor	16 (19)	8 (24)	3 (16)	5 (16)	

Tumor size, cm					0.333
Mean (SD)	20 (16)	23 (16)	17 (10)	28 (14)	

Positive lymph nodes, *n* (%)	21 (25)	13 (39)	4 (21)	4 (13)	**0.021**

Lymph node ratio					0.950
≤ 0.1	72 (86)	28 (85)	16 (84)	28 (88)	
> 0.1	11 (13)	5 (15)	3 (16)	3 (9)	

Perineural invasion, *n* (%)^g^	48 (57)	21 (64)	8 (42)	19 (59)	0.144

Perivascular invasion, *n* (%)^h^	18 (21)	8 (24)	4 (21)	6 (19)	0.683

Treatment response, *n* (%)^i^					0.307
Complete (grade 0)	5 (6)	0 (0)	2 (11)	3 (9)	
Extensive (grade 1)	27 (32)	9 (27)	7 (37)	11 (34)	
Moderate (grade 2)	33 (39)	18 (54)	6 (32)	9 (28)	
Poor (grade 3)	13 (16)	4 (12)	3 (16)	6 (19)	

Adjuvant chemotherapy, *n* (%)	38 (45)	18 (55)	7 (37)	13 (41)	0.817
Months of adjuvant administered					
Median (IQR)	3 (2–4)	3 (2–4)	2 (1–4)	4 (2–5)	0.182

Adjuvant radiotherapy, *n* (%)	7 (8)	2 (6)	2 (11)	3 (9)	0.922
Modality, *n* (%)					
SBRT	7 (8)	2 (6)	2 (11)	3 (9)	0.922

Recurrence, *n* (%)	40 (48)	12 (36)	10 (53)	18 (56)	0.111
Recurrence site, *n* (%)					0.157
Local	13 (33)	5 (15)	1 (5)	7 (22)	
Liver only	9 (23)	4 (12)	3 (16)	2 (6)	
Carcinomatosis	7 (18)	1 (3)	3 (16)	3 (9)	
Multiple site	7 (18)	1 (3)	3 (16)	3 (9)	
Lung	4 (10)	1 (3)	0 (0)	3 (9)	

LAPC: locally advanced pancreatic cancer; DP-CAR: distal pancreatectomy with celiac artery resection; IQR: interquartile range; SD: standard deviation; SBRT: stereotactic body radiation therapy. Values in bold are statistically significant.

**Table 3:** Johns Hopkins LAPC Score

LAPC score	N (%)	Celiac Artery	Superior Mesenteric Artery	Common Hepatic Artery	Portal/Superior Mesenteric Vein
**LAPC-1**	18 (27)	Encased* with GDA uninvolved	Uninvolved or abutted	Uninvolved or abutted	Uninvolved, abutted, encased* but reconstructible
	37 (55)	Uninvolved or abutted	Encased*	Uninvolved or abutted	Uninvolved or abutted
	12 (18)	Uninvolved or abutted	Uninvolved or abutted	Encased*	Uninvolved, abutted, encased* but reconstructible
**LAPC-2**	16 (27)	Encased* with GDA abutted	Uninvolved or abutted	Uninvolved or abutted	Uninvolved or abutted
	44 (73)	Uninvolved or abutted	Encased*	Uninvolved or abutted	Uninvolved, abutted, encased* but reconstructible
**LAPC3**	29 (10)	Encased* with Right sided pancreatectomy	Uninvolved abutted or encased*	Uninvolved, abutted or encased*	Uninvolved, abutted, encased* but reconstructible, non-reconstructible**
	22(8)	Encased* with GDA encasement	Uninvolved abutted or encased*	Uninvolved, abutted or encased*	Uninvolved, abutted, encased* but reconstructible, non-reconstructible**
	147 (51)	Uninvolved or abutted	Encased*	Uninvolved or abutted	Non-reconstructible**
	42 (15)	Uninvolved or abutted	Uninvolved or abutted	Encased*	Non-reconstructible**
	13 (5)	Uninvolved or abutted	Uninvolved or abutted	Proper hepatic artery involvement	Uninvolved, abutted, encased* but reconstructible, non-reconstructible**
	35 (11)	Uninvolved or abutted	Uninvolved or abutted	Uninvolved or abutted	Non-reconstructible**

* Encased: >180 degree involvement

** Non-reconstructible: (1) long segment encasement with narrowing/occlusion, (2) involvement of SMV within less than 10mm from first order tributaries, and/or (3) cavernous transformation of the PV

## Synopsis:

There is a lack of consensus management for locally advanced pancreatic cancer (LAPC). We generated a LAPC score subclassifying this broad cohort into three tiers (LAPC-1, LAPC-2, LAPC-3) based on anatomic criteria that predicts the likelihood of surgical resection.
