# Supplementary figures and images for: Anatomic Criteria Determine Resectability in Locally Advanced Pancreatic Cancer
Source: Ann Surg Oncol. Author manuscript; Available in PMC 2023 Jan 1. (PMC8688211; doi:10.1245/s10434-021-10663-1)

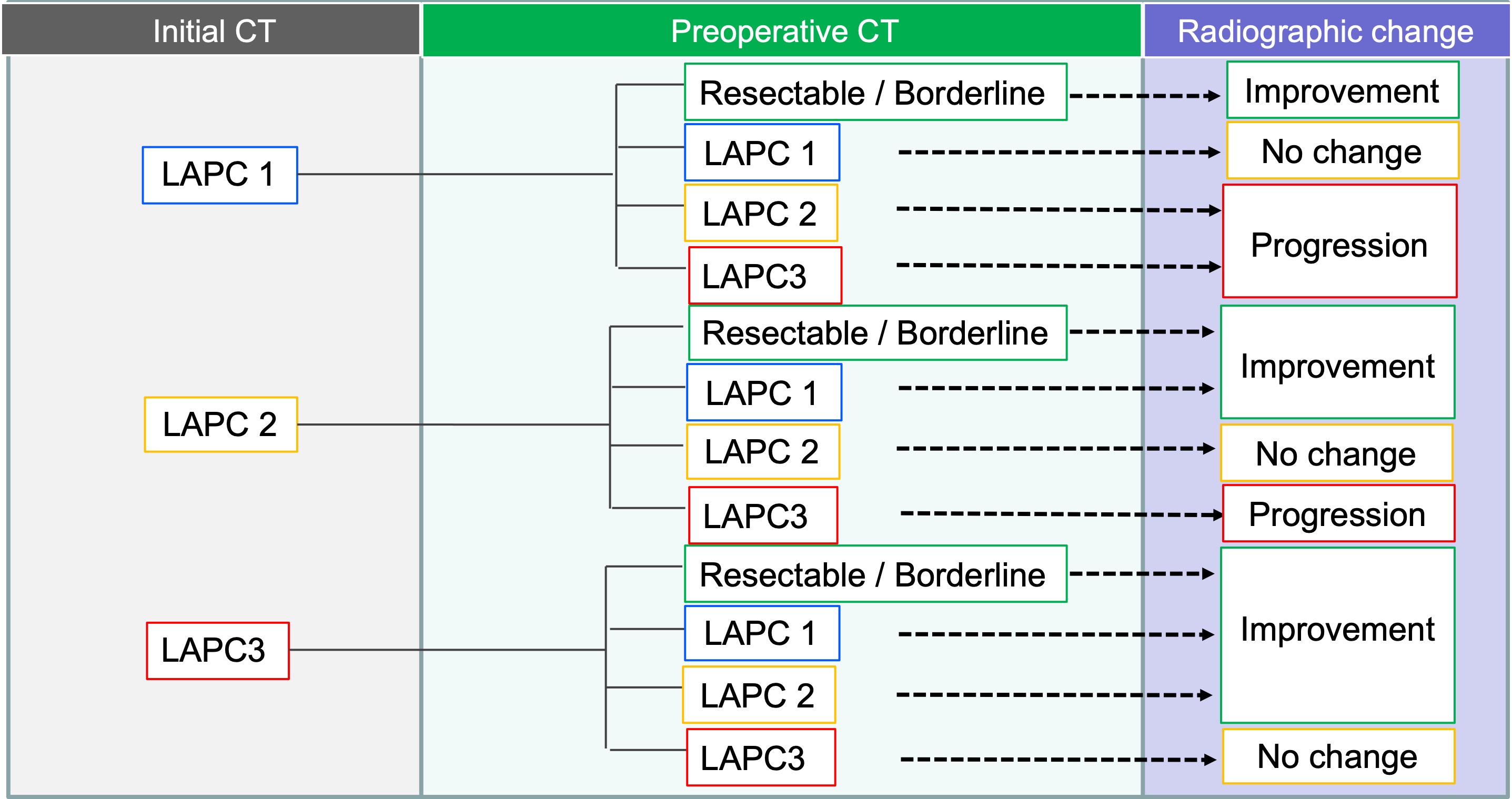

Supplement: 1741840_Sup_fig — Supplementary Figure 1: Flow chart defining radiographic change in LAPC score after neoadjuvant therapy. [file NIHMS1741840-supplement-1741840_Sup_fig.jpg]
